# On Bayesian problem-solving: helping Bayesians solve simple Bayesian word problems

**DOI:** 10.3389/fpsyg.2015.01141

**Published:** 2015-08-10

**Authors:** Miroslav Sirota, Gaëlle Vallée-Tourangeau, Frédéric Vallée-Tourangeau, Marie Juanchich

**Affiliations:** ^1^Department of Psychology, Kingston UniversityLondon, UK; ^2^Department of Management, Kingston UniversityLondon, UK

**Keywords:** Bayesian problem-solving, Bayesian research paradox, natural frequencies, Bayesian reasoning, mathematical problem solving

## Resolving the “Bayesian paradox”—Bayesians who failed to solve Bayesian problems

A well-supported conclusion a reader would draw from the vast amount of research on Bayesian inference could be distilled into one sentence: “People are profoundly Bayesians, but they fail to solve Bayesian word problems.” Indeed, two strands of research tell different stories about our ability to make Bayesian inferences—our ability to infer posterior probability from prior probability and new evidence according to Bayes's theorem. People see, move, coordinate, remember, learn, reason and argue consistently with complex probabilistic Bayesian computations, but they fail to solve, computationally much simpler, Bayesian word problems.

On the one hand, a first strand of research shows that people are profoundly Bayesians. Strong evidence indicates that the brain represents probability distributions and certain neural circuits perform Bayesian computations (Pouget et al., 2013). Bayesian computation models account for a wide range of observations on sensory perception, motoric behavior and sensorimotor coordination (see Chater et al., 2010; Pouget et al., 2013). Bayesian computations approximate observed patterns in inductive reasoning, memory, language production, and language comprehension (Chater et al., 2010). Even 12-month-old preverbal infants present behavior consistent with the behavior of a Bayesian ideal observer: infants integrate multiple sources of information to form rational expectations about situations they have never encountered before (Téglás et al., 2011). In everyday life, people form cognitive judgments predicting the occurrence of everyday events consistent with a Bayesian ideal observer (Griffiths and Tenenbaum, 2006).

On the other hand, however, a second strand of research shows that people fail to make the simplest possible Bayesian inference once they are presented with Bayesian word problems. Indeed, people tend to largely ignore or neglect base-rate information in probability judgment tasks such as social judgment or textbook problem tasks (Kahneman and Tversky, 1973; Bar-Hillel, 1980) or they tend to fail to be Bayesians in a completely opposite way—by overweighting base-rate information (Teigen and Keren, 2007). In fact, people require costly and intense training with most statistical formats to achieve good performance with probabilistic inferences that deteriorates with time very quickly (Sedlmeier and Gigerenzer, 2001).

So people are Bayesians who fail to solve simple Bayesian word problems. As with most paradoxes, a solution to this “Bayesian paradox” lies in taking closer look at conceptualizations: at what constitutes a Bayesian inference in these two strands of research. Such analysis uncovers important design differences, Bayesian classification criteria and statistical approaches (Vallée-Tourangeau et al., 2015). However, the crucial difference that we highlight here lies in the cognitive processes involved in performing the task. What is described as a “Bayesian inference” in the two strands conflates very different processes. *Implicit processes*—implicit calculations with probabilities mostly acquired from experience—are involved in the Bayesian computations approximating the performance of various cognitive functions and in the estimation of experienced real-life outcomes. *Explicit processes*—explicit calculations with probabilities typically extracted from a textual description—are involved in solving Bayesian textbook problems or social judgment problems. The different information source, experience or description, for example, has been shown to lead to dramatically different choices and decisions (e.g., Hertwig et al., 2004). With this distinction, of course, we do not intend to imply that all the cognitive processes involved in estimating probabilities are necessarily implicit and engage only with the probabilities from experience or vice versa. Rather we wish to point out that the different experimental paradigms outlined here require typically different cognitive processes operating over different types of information.

This postulated distinction between cognitive processes involved in these different types of Bayesian inference tasks can be mapped onto a distinction between biologically primary (pan-cultural, evolutionary purposeful) cognitive abilities and biologically secondary (culturally specific) cognitive abilities (Geary, 1995). It could also be linked to the debate on how people form probability judgments, either through automatic frequency encoding of sequentially presented information (e.g., Hasher and Zacks, 1984) or through heuristic inferences from aggregated information (e.g., representativeness heuristic, Kahneman and Tversky, 1974).

Which type of evidence should we call upon to help us decide whether people are Bayesians or not? Both implicit and explicit processes are relevant for assessing this ability. Having Bayesian eyes, hands and minds is arguably important for survival. Yet, our environment has changed dramatically in the Twentieth century—it became crowded with explicit aggregated statistical information. Learning from described aggregated information condenses the learning process compared with learning from experience. Imagine, for example, an experienced UK physician relocating to Nigeria. Her experience would provide her with an adequate knowledge of the disease base rates, sensitivity and specificity of medical tests within the UK population; however her experience may not be applicable or may even be deleterious in Nigeria given that those pieces of information may differ. The doctor would greatly benefit from reading explicit aggregated statistical information on base rates of diseases, sensitivity and specificity of medical tests in the local population to avoid making errors and the long learning process based on personal experience. Most importantly, she should be able to integrate this information into her diagnostic judgments when facing a given set of symptoms in a patient in Nigeria. More generally, in their probability-laden environment, all people (not just physicians) may come across a lot of problems similar to Bayesian textbook problems, of which cancer or prenatal screening are just examples (e.g., Navarrete et al., 2014). It is clear, therefore, that we should focus on improving the explicit processes that underpin Bayesian reasoning as a problem-solving ability.

## Bayesian problem-solving

Although the processes involved in solving Bayesian textbook problems resemble the processes involved in solving other mathematical problems, research on Bayesian reasoning has evolved in parallel to the research on problem solving. Reframing processes involved in Bayesian textbook reasoning in terms of the processes examined in the problem-solving literature can benefit Bayesian reasoning research efforts. The problem-solving literature not only extends the sound methodological toolkit to explore underpinning mental processes (e.g., thinking aloud protocols), but it also offers alternative concepts enacting novel insights, different explanations and more elaborate models generating deeper understanding of Bayesian problem-solving. We outline three examples of such theoretical benefits in the context of facilitating Bayesian problem-solving.

First, applying problem-solving concepts to Bayesian reasoning offers a novel and productive perspective. For example, we could think of Bayesian textbook problems in a problem-solving framework as a combination of insight and analytical problems. Typically, the problem-solving literature distinguishes two classes of problems: analytical and insight problems (Gilhooly and Murphy, 2005). With analytical problems, people can work out an incremental solution and rarely experience an Aha! moment in the process. Consider, for instance, this multi-digit addition problem: “Sum up the following numbers: 13, 27, 12, 32, 25, 11”; participants announcing an answer rarely do so with Eureka glee (although they might experience relief). With insight problems, people have to overcome an initial impasse to reach a completely new way of thinking about the problem; they need to transform the initial problem representation into a new representation which will lead them to the goal state. Consider, for instance, the following problem: “Place 17 animals in 4 enclosures in such a manner that there will be an odd number of animals in each enclosure” (adapted from Metcalfe and Wiebe, 1987). You probably try 17/4 and it did not work: The problem masquerades as an arithmetic puzzle. However, in contrast to an analytic problem, the initial problem presentation cannot be transformed step-by-step to a solution (in this case the solution involves overlapping sets). This distinction suggests that decomposing the question of “What facilitates Bayesian reasoning?” into “What facilitates the insight?” and “What facilitates the computation?” will pave the way for better understanding what factors facilitate the problem-structure understanding and what factors facilitate the computational operations in Bayesian problem-solving (see also Johnson and Tubau, 2015).

Second, rephrasing Bayesian reasoning as a form of problem-solving offers different explanations of the processes implicated, for example, those involved in representational training (e.g., Sedlmeier and Gigerenzer, 2001; Mandel, 2015; Sirota et al., 2015a). In representational training, participants learn to transform the statistical format representation of a problem—they learn to translate single-event probabilities into natural frequencies. For example, the statements “a 1% probability that a woman has breast cancer” and “if a woman has cancer then there is an 80% probability that she will get a positive mammogram” are translated as “10 out of every 1000 women have breast cancer” and “8 out of the 10 who have breast cancer will get a positive mammogram.” The problem-solving approach posits that the underlying mechanism of such representational training consists of the acquisition of an appropriate problem representation—a nested-sets representation of the Bayesian problem, regardless of frequencies or probabilistic information contained in such problem—during the learning phase, which is then transferred to similar problems in the testing phase (for evidence see Sirota et al., 2015a). This goes beyond the default explanation that participants translated single-event probabilities into natural frequencies (Sedlmeier and Gigerenzer, 2001) and it accounts for the training success in terms of the specific mental processes involved in problem representation learning and its transfer (for the importance of a good representation in different problems of a belief revision not depending on natural frequencies, see Mandel, 2014).

Third, recruiting problem-solving models offers a better understanding of well-known effects in Bayesian reasoning than we currently have, for example, the format effect. Statistical formats such as natural frequencies represent probably the most cost-effective (and the most discussed) tool to facilitate Bayesian problem-solving, given that visual aids offer mixed evidence of their effectiveness (e.g., Cosmides and Tooby, 1996; Sirota et al., 2014b). Natural frequencies enhance Bayesian problem-solving when compared with formats involving normalization such as probability formats (e.g., Gigerenzer and Hoffrage, 1995; Cosmides and Tooby, 1996; Barbey and Sloman, 2007). Natural frequencies, introduced by Kleiter (1994), integrate the base-rate information in their structure making the base-rate information per se redundant. For example, the statement “8 women out of the 10 who have breast cancer will get a positive mammogram” includes the base-rate information of *the* 10 (out of 1000) women with cancer from our previous example.

According to the general framework of mathematical verbal problem solving (Kintsch and Greeno, 1985; Kintsch, 1988), which integrates formal mathematical and linguistic knowledge, two processes should be differentiated here: the processes involved in representing the problem and those involved in producing a solution (for specific approaches to probability representation, see Johnson-Laird et al., 1999; Mandel, 2008). In the problem representation phase, a mental representation is constructed from the text that triggers available knowledge schemas stored in long-term memory. Familiar cues in the text activate a correct mental representation of the problem more easily than unfamiliar or misleading ones; this enables an easier integration with existing knowledge. In the problem solution phase, rules or strategies corresponding to the problem representation are implemented. We suggest that the facilitative effect of natural frequencies in Bayesian inference problems is due to a similar process. A wording of the task with frequencies (e.g., explicit set reference language such as “10 *out of* the *remaining* 90”)—not the numerical format by itself—may trigger a representation of the problem as nested sets, while a wording of the task with probabilities which conceal the nested set structure due to normalizing, does not. Such an explanation casts natural frequencies as a familiar format rather than a privileged one. Some authors view natural frequencies as a privileged format because they are processed by a specialized frequency-coding mechanism shaped by evolutionary forces (Gigerenzer and Hoffrage, 1995). If true (and some specific conditions are fulfilled, Barrett et al., 2006) then processing of a privileged format should not be cognitively demanding at all or at least less cognitively demanding than processing of a computationally equivalent and equally familiar format (e.g., Cosmides and Tooby, 1996). It means, for instance, that measures of cognitive capacity should not be predictive of performance in Bayesian reasoning. However, several recent studies have provided evidence rebutting the claim of easier processing of natural frequencies (Sirota and Juanchich, 2011; Lesage et al., 2013; Sirota et al., 2014a).

## Conclusion

Our environment is laden with statistical information and demands from people that they successfully solve problems that are exactly the same as, or similar to, classical Bayesian textbook problems. Although some brain function appears to implement Bayesian computations, people's abilities to solve Bayesian word problems could still be substantially improved. We should therefore strive to understand and improve people's performance with this kind of problems. We suggest thinking about the involved processes as processes akin to those engaged during problem-solving (see also Johnson and Tubau, 2015; Sirota et al., 2015b). Such a re-classification would not only resolve contradictions in research on Bayesian inference, it would also facilitate the application of conceptual and methodological tools from problem-solving research. It would allow us to ask what enacts the insight about the problem structure, what facilitates the relevant computations and how exactly people implement these processes. It would allow us to conceptually re-frame observed effects such as representational training effects. It would also allow us to shed more light on the underlying processes by utilizing elaborate process-oriented models developed in this area.

### Conflict of interest statement

The authors declare that the research was conducted in the absence of any commercial or financial relationships that could be construed as a potential conflict of interest.
